# Influence of Accelerometer Type and Placement on Physical Activity Energy Expenditure Prediction in Manual Wheelchair Users

**DOI:** 10.1371/journal.pone.0126086

**Published:** 2015-05-08

**Authors:** Tom Edward Nightingale, Jean-Philippe Walhin, Dylan Thompson, James Lee John Bilzon

**Affiliations:** Centre for DisAbility Sport and Health (DASH), Department for Health, University of Bath, Bath, Somerset, United Kingdom; Vanderbilt University, UNITED STATES

## Abstract

**Purpose:**

To assess the validity of two accelerometer devices, at two different anatomical locations, for the prediction of physical activity energy expenditure (PAEE) in manual wheelchair users (MWUs).

**Methods:**

Seventeen MWUs (36 ± 10 yrs, 72 ± 11 kg) completed ten activities; resting, folding clothes, propulsion on a 1% gradient (3,4,5,6 and 7 km·hr^-1^) and propulsion at 4km·hr^-1 ^(with an additional 8% body mass, 2% and 3% gradient) on a motorised wheelchair treadmill. GT3X+ and GENEActiv accelerometers were worn on the right wrist (W) and upper arm (UA). Linear regression analysis was conducted between outputs from each accelerometer and criterion PAEE, measured using indirect calorimetry. Subsequent error statistics were calculated for the derived regression equations for all four device/location combinations, using a leave-one-out cross-validation analysis.

**Results:**

Accelerometer outputs at each anatomical location were significantly (*p <* .*01*) associated with PAEE (GT3X+-UA; *r * = 0.68 and GT3X+-W; *r * = 0.82. GENEActiv-UA; *r* = 0.87 and GENEActiv-W; *r* = 0.88). Mean ± SD PAEE estimation errors for all activities combined were 15 ± 45%, 14 ± 50%, 3 ± 25% and 4 ± 26% for GT3X+-UA, GT3X+-W, GENEActiv-UA and GENEActiv-W, respectively. Absolute PAEE estimation errors for devices varied, 19 to 66% for GT3X+-UA, 17 to 122% for GT3X+-W, 15 to 26% for GENEActiv-UA and from 17.0 to 32% for the GENEActiv-W.

**Conclusion:**

The results indicate that the GENEActiv device worn on either the upper arm or wrist provides the most valid prediction of PAEE in MWUs. Variation in error statistics between the two devices is a result of inherent differences in internal components, on-board filtering processes and outputs of each device.

## Introduction

The positive contribution of regular physical activity (PA) to weight balance, metabolic regulation and cardiovascular fitness is well documented and broadly accepted in the able-bodied population [1]. Epidemiological studies concerning the impact of PA on health in wheelchair users with a spinal cord injury (SCI) have been limited to assessing associations between subjective reports of activities of daily living (ADL) [2] or leisure time physical activity (LTPA) [3] and chronic disease risk factors. The assessment of these constructs in previous studies were quantified via the Physical Activity Recall Assessment for people with a SCI [PARA-SCI; [4]], which is administered via a telephone interview. This subjective PA assessment tool asks participants to recall activities undertaken during the previous 3 days, which is a relatively short monitoring period. The results, like other self-report measures, depend on the accuracy of the participants’ memory and recall [5]. Ullrich *et al*, [6] also suggested that the use of the PARA-SCI might have limited application for other investigators besides the developers due to the exclusion of subjective appraisals and the technical complexity of interview administration. To date, despite the aforementioned limitations, quantifying free living PA among wheelchair users has mostly been restricted to self-report measurements. As such there is a requirement to develop unobtrusive objective measurement tools that can be easily used to characterise the association between physical activity and metabolic health in this population.

The PA monitoring literature has evolved rapidly, particularly in able-bodied populations, yet there is a paucity of research focussing on the use of activity monitors to predict physical activity energy expenditure (PAEE) in certain populations, including manual wheelchair users (MWUs). Broadly speaking, various devices used previously to determine PA in MWUs have distinct limitations. For example despite being unobtrusive, a tri-axial accelerometer attached to a wheelchair [7] is unable to distinguish between self or assisted propulsion and is also unable to quantify any activity out of the wheelchair, or during stationary arm crank ergometry exercise. Monitors attached on the waist near the participants’ centre of mass, as advised by manufacturers for use in able-bodied cohorts, have been shown to under-estimate energy expenditure by 24% in MWUs [8]. Previous work by our research group [9] and others [10] has indicated that the anatomical placement location is critical to accurately estimate PAEE. It is perhaps not surprising due to restricted patterns of movement that a tri-axial accelerometer worn on the wrist explains more of the variance in PAEE, resulting in the lowest random error compared to the waist. The use of multi-sensor devices has mostly been limited to laboratory-based validation of the SenseWear Armband (SWA). With the development of specific energy expenditure (EE) prediction equations for MWUs [11] the SWA’s accuracy has been improved somewhat. However, even when using these specific prediction equations, the SWA tended to overestimate EE (27 to 43%) whereas a wrist-mounted accelerometer accurately predicted EE (9 to 25%) during wheelchair propulsion [12]. Also a recent free-living study using doubly-labelled water demonstrated that the SWA was unable to detect variation in within-individual EE during voluntary increases in physical activity in individuals with a SCI [13].

The technological advancements in the field of PAEE assessment have stimulated the development of sensitive tri-axial accelerometers, capable of storing higher resolution raw, unfiltered acceleration signals, with increased memory capacity for capturing data over prolonged periods of time [14]. Previous work validating objective PA monitoring tools in MWU’s have only reported accelerometer outputs as arbitrary count values. The GENEActiv device gives raw acceleration values, reporting signal vector magnitude (SVM) in *g*-seconds (*g*·s^-1^). This device is not subject to on-board manufacturer-defined band pass filters and hence does not demonstrate the plateau phenomenon of the GT3X+ observed previously [9]. Furthermore, it remains to be established whether fluctuations in criterion PAEE during wheelchair propulsion over differing gradients or during load carriage can be detected by accelerometer outputs at the wrist. Therefore, the aim of the present study was to assess the validity of two commonly used accelerometer devices, at two different anatomical locations, for the prediction of PAEE in MWUs in a controlled laboratory environment.

## Methods

Ethics approval was granted by the University of Bath Research Ethics Approval Committee for Health (REACH) and written informed consent was obtained from each participant. Seventeen (n = 17) male manual wheelchair users visited the Centre for DisAbility Sport and Health (DASH) human physiology laboratory on one morning following an overnight fast. Ten of the participants had complete paraplegia with lesion levels ranging from T1 to L4. Other conditions responsible for use of a wheelchair included Spina Bifida (n = 3), Cerebral Palsy (n = 1) and Scoliosis (n = 1). A bilateral lower limb amputee (n = 1), who was considered a regular wheelchair user (>70% of locomotion manual wheelchair propulsion) and an able-bodied wheelchair basketball player (n = 1) who was also familiar with wheelchair propulsion (> two years) were also included in the analysis. Previous work from our research group demonstrated that the inclusion of numerous disabilities had no measurable impact on the prediction of PAEE in MWUs [9]. Other research has also included participants with various aetiologies responsible for wheelchair use when assessing methods to predict EE in this population [15]. If anything, this approach provides a robust model for the assessment of PAEE in the broader MWU population and the inclusion of a diverse range of participants is in accordance with best practice recommendations for PA validation studies [16].

Time since injury (TSI) was self-reported based on when the medical condition was first diagnosed by a clinician. The mass of the wheelchair and participant was recorded in light clothing to the nearest decimal place using platform wheelchair scales (Detecto BRW1000, Missouri, USA). The wheelchair, along with participants shoes were weighed separately and subtracted from the total mass of the participant plus wheelchair to derive an accurate body mass (kg) [17]. Waist and hip circumference (cm) were measured in duplicate, with participants lying flat on a hard physiotherapy bed, using a metallic tape measure (Lufkin, US). The mean value was taken, and waist/ hip ratio calculated. Skinfold thickness (mm) at 4 upper body sites (biceps, triceps, subscapular, suprailiac) were measured in duplicate using a set of skinfold calipers (Holtain Ltd, Crymych, UK); the mean value was calculated. Resting metabolic rate) (RMR; kcal·day^-1^) was measured in a semi-recumbent position in accordance with best practice [18] using a TrueOne 2400 computerized metabolic system (ParvoMedics, Salt Lake City, UT). Blood pressure (mmHg) was also measured using an automated blood pressure monitor (Boso Medicus Prestige, Bosch + Sohn, Germany) in a semi-recumbent positions following RMR. Three readings were taken and the mean value was calculated.

### Activity Protocol

Following the measurement of RMR and anthropometric assessment, participants performed an activity protocol, which consisted of wheelchair propulsion and a folding clothes task, representative of an activity of daily living. Participants continuously untangled t-shirts placed on a desk, then neatly folded and stacked them. Wheelchair propulsion took place on a wheelchair adapted treadmill with necessary safety features and stabilising arm (HP Cosmos Saturn 250/100r, HaB International Ltd, UK). A counterbalanced approach for randomly assigning the order of activities was not utilised in this study based on observations from previous work [9]. Even with five minutes of recovery in between activities, a considerable carryover effect in oxygen uptake (V̇O_2_) and heart rate (HR) was observed. Therefore, each activity was assigned in order of intensity and lasted for six minutes interspersed with four minute recovery periods. Wheelchair propulsion on the adapted treadmill included eight trials 3 km·hr^-1^, 4 km·hr^-1^, 5 km·hr^-1^, 6 km·hr^-1^ & 7 km·hr^-1^ on a 1% gradient. Following a ten minute rest, participants propelled at 4 km·hr^-1^ on a 1% gradient with 8% of body mass added to the chair in a rucksack and 4 km·hr^-1^ on a 2% and 3% gradient.

### Instrumentation and assessment of energy expenditure

The GT3X+ activity monitor (Actigraph, Pensacola, FL, USA) records time-varying accelerations within the dynamic range of ± 6 *g*, and contains a solid state tri-axial accelerometer. The GT3X+ activity monitor is compact (dimensions: 4.6 cm x 3.3 cm x 1.9 cm), lightweight (19 grams), and can easily be worn at multiple locations on the body. To quantify the amount and frequency of human movement, accelerometer outputs are digitized via a twelve-bit analog to digital converter (A/DC) and passed through Actigraph’s proprietary digital filtering algorithms. In order to eliminate any acceleration noise outside of the normal human activity frequency, digitized signals pass through low (0.25 Hz) and high (2.5 Hz) band width filters [19]. ‘Physical activity counts’ (PAC) are calculated through summing the change in raw acceleration values measured during a specific interval of time, or ‘epoch’. The GENEActiv tri-axial device (GENEActiv, Activinsights, Cambridge, UK) contains a ± 8 *g* seismic accelerometer and is also compact (dimensions: 4.3 cm x 4.0 cm x 1.3 cm) and lightweight (16 grams). This device has been explained in detail elsewhere [20]. Both devices were initialised with a sampling frequency of 30 Hz.

Throughout the activity protocol two GT3X+ units were worn, one on the right wrist (W, using a Velcro wrist strap positioned over the dorsal aspect of the wrist midway between the radial and ulnar Styloid processes) and one on the right upper arm (UA, using a small elastic belt positioned on the lateral surface of the arm midway between the acromion process and lateral epicondyle of the humerus). Two GENEActiv accelerometers were also worn; one distal to the GT3X+ on the right W and one on the posterior aspect of the midpoint on the right UA, securely fixed to the skin over the triceps brachii using a 10 cm^2^ patch of tape (Hyperfix self-adhesive dressing retention tape, Smith & Nephew Healthcare Ltd., UK). The GENEActiv and GT3X+ devices were both initialised with a sampling frequency of 30Hz.

Continuous gas exchange measurements were collected during each activity, using a TrueOne 2400 computerized metabolic system,calibrated according to manufacturer’s instructions prior to use. Fractions of oxygen and carbon dioxide were measured via a paramagnetic oxygen analyser and an infrared, single beam, single wave-length carbon dioxide analyser, respectively. Metabolic data was retrieved and analysed using associated software (TrueOne metabolic software, Salt Lake City, UT). V̇O_2_ and carbon dioxide production (V̇CO_2_) were used to estimate EE (kcal^.^min^-1^) of each activity, using indirect calorimetry. A Polar Team HR monitor (Polar Electro Inc., Lake Success, NY, USA) was also worn during each activity. Accelerometer outputs and physiological variables for each task and each participant can be found in the supporting information file (S1 Dataset). Peak oxygen uptake (V̇O_2_ peak) was determined at the end of the activity protocol using a continuous, progressive intensity test with three minute exercise stages until the point of volitional exhaustion. This was conducted using an electrically braked arm crank ergometer (Lode Angio, Groningen, Netherlands) using a prescribed crank rate of 70 rev·min^-1^.

### Statistical Analyses

An *a priori* power calculation revealed a sample size of fifteen was necessary in order to detect a correlation coefficient (*r)* of 0.67 using a one-tailed test with an α = 0.05 and power = 0.95. This calculation was based on activity count (counts·s^-1^) from a Computer Science and Applications (CSA) accelerometer and V̇O_2_ data during a protocol which included three wheelchair propulsion velocities [21]. Activity monitors were synchronised prior to use. Breath-by-breath data was exported into Microsoft Excel from the TrueOne metabolic software and averaged over the final two minutes of each activity. Assuming that dietary-induced thermogenesis was negligible (participants came into the laboratory following a 10-hr overnight fast) resting metabolic rate (kcal·min^-1^) was subtracted from total energy expenditure (TEE) measured by the TrueOne 2400 computerized metabolic system to generate PAEE for each activity. Comparisons between the ‘criterion’ measurement of PAEE [TEE—RMR] and activity monitors were made between the final two minutes of each activity.

#### Data Modelling

GT3X+ and GENEActiv units were downloaded using ActiLife software (Actigraph, Pensacola, FL, USA) and GENEActiv PC software (version 1.2.1, Activinsights, Cambridge, UK), respectively. Data was exported to Microsoft Excel in a time and date stamped comma-separated value (c.s.v.) file format. Physical activity counts (counts·min^-1^) from the GT3X+ and Signal vector magnitude (SVM_*g*s_) data from the GENEActiv were summated into 60-s epochs. Activity counts (counts·min^-1^) from the GT3X+ and raw acceleration values (*g*·min^-1^) from the GENEActiv were then averaged over the final two minutes of each activity. Physical activity energy expenditure prediction models were developed using corresponding data from each task for each device at each location, using linear regression analysis. The dependent variable was PAEE (kcal·min^-1^) during the final two minutes of each task (that is, 171 values in total). The independent variables included accelerometer outputs, i.e. counts·min^-1^ and SVM_*g*_._min-1_ for the GT3X+ and GENEActiv, respectively. Pearson product moment correlation coefficients (*r*) and coefficients of determination (R^2^) statistics were conducted to assess the association between the criterion PAEE and outputs from each device at each location. Standard Error of the Estimate (SEE) was also calculated for each correlation.

#### Error statistics

When the development and evaluation of predictive models are conducted on the same participants, subsequent evaluation statistics tend to be biased and are often overly optimistic [22]. Hence, there is a need to cross-validate generated prediction equations using an independent sample; this can be achieved by using a ‘split sample’ approach whereby half of the participants are used for developing the models and half for cross-validation. However, it is not always feasible to utilise this ‘split sample’ approach when sample sizes are small, a common occurrence when working with disabled populations due to challenges with participant identification and recruitment [23]. This problem was overcome by developing the estimation algorithm on all but one of the participants and then evaluating it on the ‘held-out’ participant by calculating the PAEE prediction error. This process was repeated where each participant acted as the held-out participant and the mean of all the error evaluations were calculated. This procedure is a ‘leave-one-out cross validation’ described in more detail elsewhere [24].

The comparison statistics involved calculating the mean absolute error (MAE) and mean signed error (MSE) for each activity, the later will be displayed graphically using modified Bland and Altman plots. Considering it is likely the absolute error of estimation will increase with exercise intensity [22], error of estimate data will also be presented as a percentage. A two-way mixed model ANOVA was performed on the raw data to determine differences between criterion PAEE and predicted PAEE. Where a significant interaction effect was observed, a Ryan-Holm-Bonferroni Stepwise Adjustment was applied to *post hoc* tests where multiple comparisons were considered. This was to identify the specific activities in which there were significant differences between the criterion and predicted PAEE. Statistical significance was set at *a priori* of α < 0.05. All analyses were performed using IBM SPSS Statistics 20 for Windows (IBM, Armonk, NY, USA).

## Results

Demographic and anthropometric characteristics of the participants are described in Table 1. Criterion PAEE (kcal·min^-1^) and accelerometer outputs at each anatomical location are displayed in Table 2. Four [SCI (T1 and T2); n = 2 Cerebral Palsy; n = 1 and Scoliosis; n = 1] and two participants (T1 and Cerebral Palsy) were unable to complete the 7km·hr^-1^ propulsion speed and 4km·hr^-1^ (3% gradient) tasks, respectively. PAEE, HR and rating of perceived exertion (RPE) increased with increasing velocity of wheelchair propulsion and during steeper gradients. Calculated metabolic equivalents (METs) from dividing V̇O_2_ for each activity by individual V̇O_2_ determined at rest, suggests that all the propulsion trials besides 3km·hr^-1^were on average considered as moderate-intensity activities, whereas folding clothes and propulsion at 3km·hr^-1^ were light-intensity activities.

**Table 1:** Demographic and anthropometric characteristics of the participants.

Variable	Mean ± SD	Range (lowest—highest)
Age (years)	36 ± 10	19–50
Body mass (kg)	71.6 ± 10.6	53.4–87.5
Time since injury (years)	15 ± 14	2–50
Total skinfold from 4 sites (mm)	48.5 ± 20.3	24.6–110.9
Waist-hip ratio	1.0 ± 0.1	0.8–1.1
Blood pressure (mmHg)		
* Systolic*	133 ± 18	108–174
* Diastolic*	84 ± 16	62–116
RMR (kcal·day^-1^)	1571 ± 254	1201–2152
V̇O_2_ peak (ml·kg^-1^·min^-1^)	27.0 ± 8.0	16.7–41.1

Note: Able-bodied participant was not included in Time since injury

**Table 2:** Measured PAEE, accelerometer outputs at each anatomical location, calculated METs, heart rate, RPE and number of participants per trial for each activity (mean ± SD).

Activity	Measured PAEE (Kcal·min^-1^)	Physical activity counts·min^-1^		SVM (*g*·min^-1^)		MET (calculated)	Heart rate (b·min^-1^)	RPE	n
		GT3X+-UA	GT3X+-W	GENEActiv-UA	GENEActiv-W				
**Resting**	0.0 ± 0.0	46 ± 57	119 ± 151	30 ± 16	46 ± 24	1.0 ± 0.0	65 ± 12	-	17
**Folding clothes**	1.1 ± 0.2	3843 ± 1235	8905 ± 1885	121 ± 22	296 ± 66	2.0 ± 0.3	85 ± 15	8 ± 2	17
**3 km·hr** ^**-1**^	1.9 ± 0.4	7008 ± 1751	8806 ± 1973	330 ± 78	529 ± 125	2.7 ± 0.4	90 ± 13	9 ± 2	17
**4 km·hr** ^**-1**^	2.4 ± 0.6	7056 ± 1761	10283 ± 2569	421 ± 110	708 ± 220	3.2 ± 0.6	97 ± 20	10 ± 3	17
**5 km·hr** ^**-1**^	3.1 ± 1.0	7100 ± 1405	12066 ± 4382	492 ± 127	898 ± 353	3.8 ± 0.8	114 ± 23	12 ± 3	17
**6 km·hr** ^**-1**^	4.2 ± 1.7	7615 ± 1422	14918 ± 5500	618 ± 154	1170 ± 491	4.7 ± 1.2	130 ± 33	14 ± 3	17
**7 km·hr** ^**-1**^	4.7 ± 0.9	8602 ± 1898	16367 ± 5492	701 ± 151	1244 ± 355	5.1 ± 0.8	136 ± 26	14 ± 3	13
**4 km·hr** ^**-1**^ **(+ 8% of body mass)**	2.5 ± 0.7	7151 ± 2091	10193 ± 2718	397 ± 106	667 ± 240	3.4 ± 0.6	111 ± 20	10 ± 3	17
**4 km·hr** ^**-1**^ **(2% gradient)**	3.2 ± 0.8	7477 ± 1891	10934 ± 3503	455 ± 109	760 ± 276	3.9 ± 0.7	119 ± 24	12 ± 3	17
**4 km·hr** ^**-1**^ **(3% gradient)**	4.0 ± 0.9	7852 ± 1785	11439 ± 2686	512 ± 121	830 ± 223	4.6 ± 0.9	128 ± 22	13 ± 4	15

### Data Modelling

**Fig 1:**
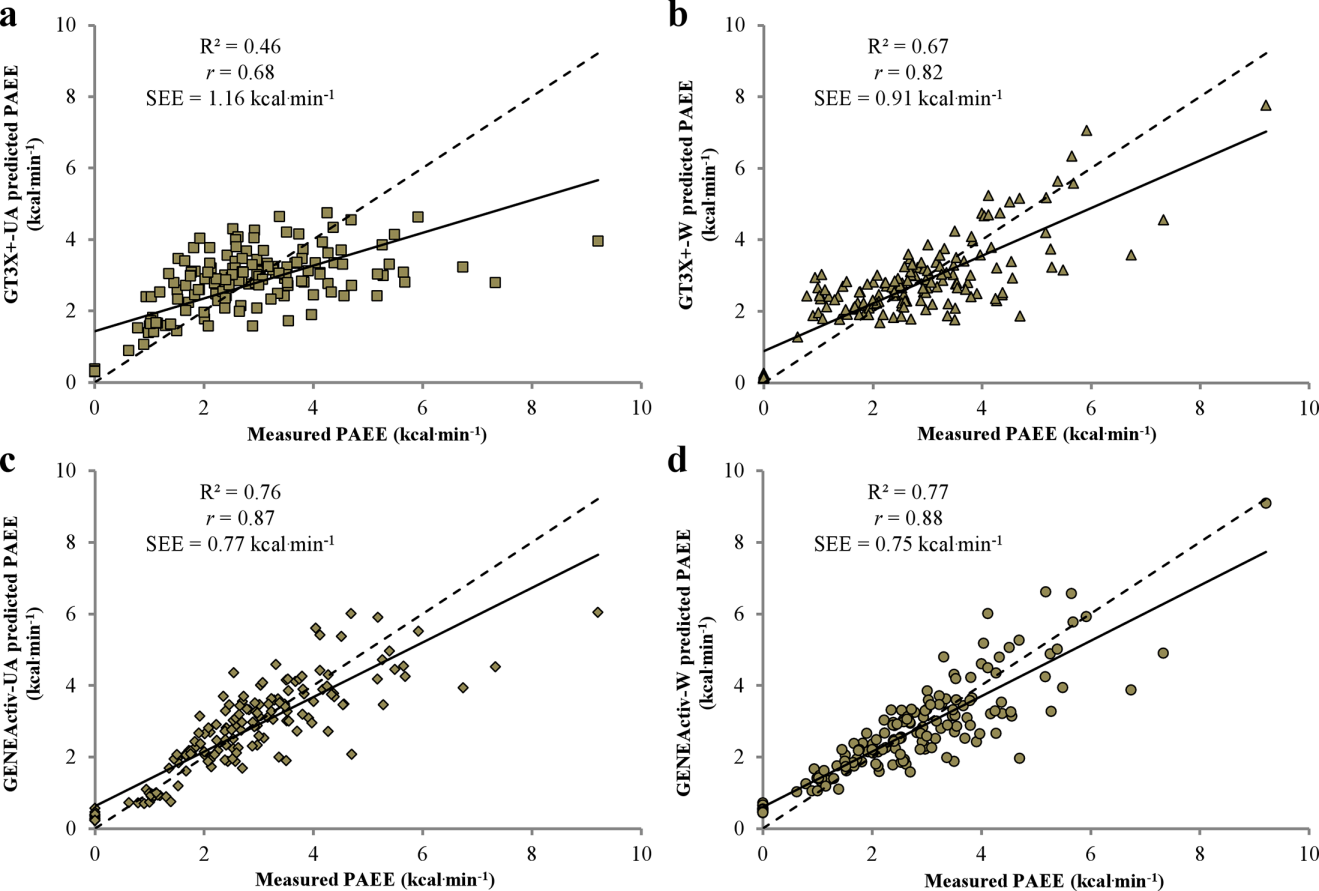
Scatterplots showing the relationship between predicted PAEE for the GT3X+-UA (a), GT3X+-W (b), GENEA-UA (c) and GENEA-W (d). The straight line represents the models best fit, and the dotted line indicates the line of identity.

The associations between criterion PAEE measured by the TrueOne 2400 computerized metabolic system and predicted PAEE derived from accelerometer outputs from each device are presented as scatter plots in Fig 1. Physical activity counts from the GT3X+ were significantly (*P* < 0.01) associated with PAEE (UA; *r* = 0.68, W; *r* = 0.82), as were raw acceleration outputs from the GENEActiv (UA; *r* = 0.87, W; *r* = 0.88). The SEE for the correlations were 1.16 and 0.91 kcal·min^-1^ for the GT3X+ worn at the UA and W, 0.77 and 0.75 kcal·min^-1^ for the GENEActiv worn at the UA and W. The linear regression equations using activity counts from the GT3X+ (Eq 1 and 2) and raw acceleration outputs from the GENEActiv (Eq 3 and 4) for each location are shown beneath.

$$PAEE_{UA}=(0.000372\;*\;{\text{Physical activity counts}\cdot \text{min}}^{-1})+0.291708$$ (1)

$$PAEE_{W}=(0.000245\;*\;{\text{Physical activity counts}\cdot \text{min}}^{-1})+0.132379$$ (2)

$$PAEE_{UA}=(0.006260\;*\;\text{SVM}\;g\cdot {\text{min}}^{-1})+0.139778$$ (3)

$$PAEE_{W}=(0.003210\;*\;\text{SVM}\;g\cdot {\text{min}}^{-1})+0.392209$$ (4)

### Error Statistics

**Fig 2:**
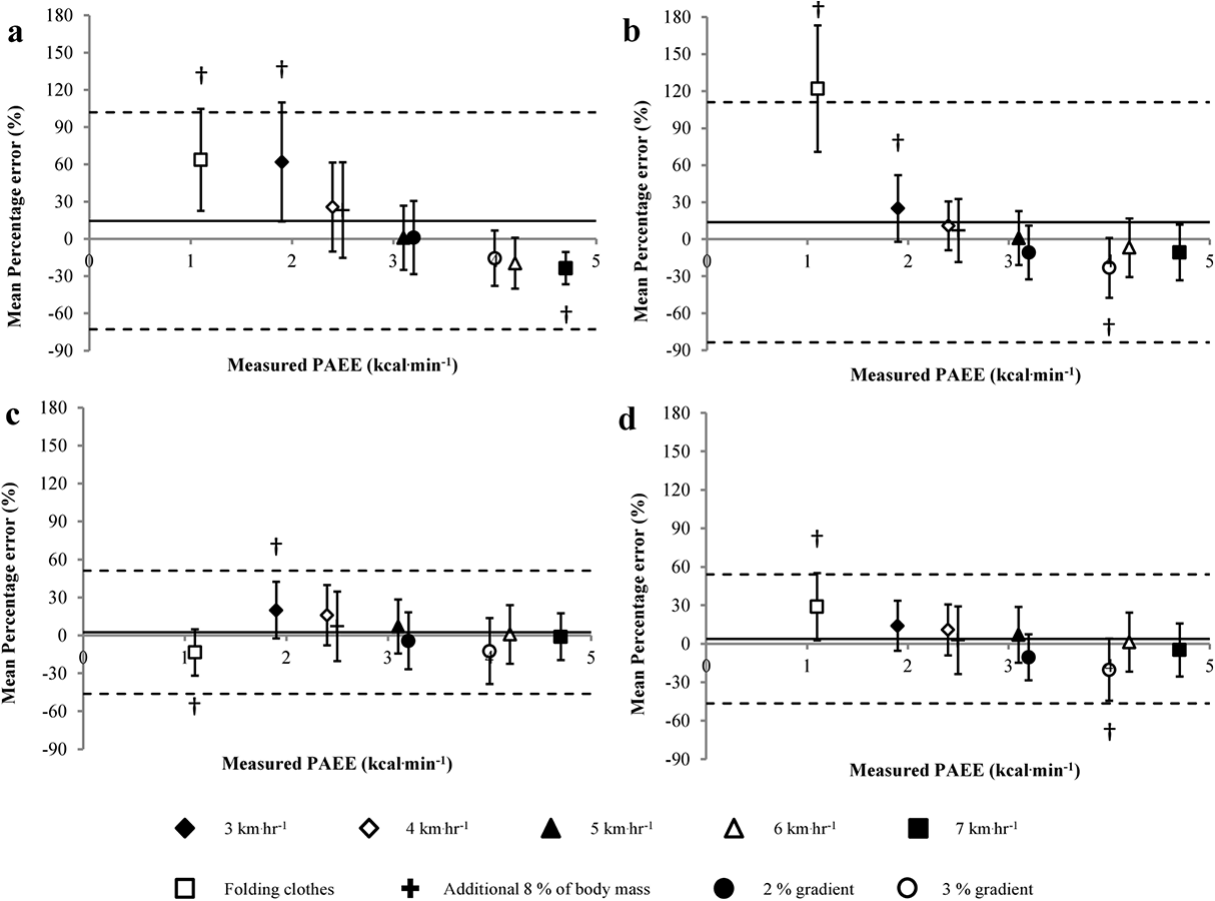
Modified Bland and Altman plots displaying error of estimated PAEE expressed as a percentage for GT3X+-UA (a), GT3X+-W (b), GENEA-UA (c) and GENEA-W (d) for each activity. The solid line indicates overall percentage error of estimate. The dotted lines indicate the upper and lower 95% LoA. † Indicates significant difference (P ≤ 0.05) from the criterion PAEE.

Modified Bland and Altman plots illustrate overall percentage error of estimate [± 95% limits of agreement (LoA)] between criterion PAEE and predicted PAEE derived from the developed linear regressions; 15 ± 87%, 14 ± 97%, 3 ± 49% and 4 ± 50% for the GT3X+-UA, GT3X+-W, GENEActiv-UA and GENEActiv-W, respectively (Fig 2). The GT3X+-W significantly (*P* < 0.05) over-predicted propulsion at 3km·hr^-1^ (mean ± SD; 25 ± 27%), as does the GT3X+-UA (62 ± 48%) and GENEActiv-UA (20 ± 22%). Both the GT3X+-W (-23 ± 24%) and GENEActiv-W (-20 ± 24%) significantly under-predicted PAEE during propulsion at 4km·hr^-1^ on a 3% gradient. The GT3X+-UA, GT3X+-W and GENEActiv-W over-predicted PAEE during a simulated folding clothes task by 64 ± 50%, 122 ± 51% and 29 ± 26%, respectively, whereas the GENEActiv-UA significantly under-predicted PAEE (-14 ± 18%). All monitors significantly over-predicted PAEE during rest by 0.31, 0.16, 0.32 and 0.54 kcal·min^-1^ for the GT3X+-UA, GT3X+-W, GENEActiv-UA and GENEActiv-W, respectively.

Table 3 shows the MAE and mean absolute percentage difference between the criterion and estimated PAEE. Absolute PAEE estimation errors varied from 19 to 66% for the GT3X+-UA, 17 to 122% for the GT3X+-W, 15 to 26% for the GENEActiv-UA and 17 to 32% for the GENEActiv-W. The aetiology responsible for wheelchair use was evaluated to see if it impacted on the fit of the model during our leave-one-out analysis. No trend with regards to increased mean absolute error for various aetiologies was observed. There was no relationship between wheelchair experience, TSI was used as a surrogate for this, and error. Furthermore, looking specifically at participants with paraplegia, there was no relationship between level of SCI lesion (indicative of function) and magnitude of error.

**Table 3:** Mean absolute error (MAE; kcal·min^-1^) and Mean absolute percentage error of predicted PAEE using generated linear regression equations for each monitor at each location.

Activity	MAE (kcal·min^-1^)				Mean absolute percentage error (%)			
	GT3X+-UA	GT3X+-W	GENEActiv-UA	GENEAcitv-W	GT3X+-UA	GT3X+-W	GENEActiv-UA	GENEAcitv-W
**Resting**	0.31 ± 0.05	0.16 ± 0.07	0.32 ± 0.11	0.54 ± 0.08	-	-	-	-
**Folding clothes**	0.66 ± 0.41	1.24 ± 0.46	0.21 ± 0.17	0.32 ± 0.19	63.7 ± 41.0	122.0 ± 51.3	18.9 ± 12.2	32.1 ± 22.0
**3 km·hr** ^**-1**^	1.15 ± 0.65	0.48 ± 0.33	0.46 ± 0.27	0.34 ± 0.22	66.1 ± 41.6	28.0 ± 23.7	25.8 ± 14.7	19.1 ± 14.2
**4 km·hr** ^**-1**^	0.86 ± 0.57	0.42 ± 0.24	0.52 ± 0.45	0.42 ± 0.29	35.9 ± 24.7	18.4 ± 12.3	21.9 ± 18.3	17.9 ± 13.5
**5 km·hr** ^**-1**^	0.67 ± 0.84	0.54 ± 0.43	0.61 ± 0.42	0.60 ± 0.42	19.3 ± 16.6	17.1 ± 12.9	18.9 ± 11.3	18.8 ± 12.2
**6 km·hr** ^**-1**^	1.14 ± 1.53	0.90 ± 0.81	0.87 ± 1.02	0.74 ± 0.75	21.7 ± 18.1	19.7 ± 14.1	17.3 ± 15.0	17.5 ± 14.4
**7 km·hr** ^**-1**^	1.13 ± 0.72	0.92 ± 0.84	0.73 ± 0.51	0.79 ± 0.67	23.6 ± 13.0	18.7 ± 16.0	15.2 ± 9.7	16.5 ± 12.8
**4 km·hr** ^**-1**^ **(+ 8% of body mass)**	0.96 ± 0.61	0.52 ± 0.31	0.59 ± 0.33	0.55 ± 0.31	38.0 ± 22.9	21.5 ± 14.5	24.1 ± 14.0	22.6 ± 12.9
**4 km·hr** ^**-1**^ **(2% gradient)**	0.81 ± 0.83	0.64 ± 0.51	0.56 ± 0.56	0.54 ± 0.46	22.9 ± 17.7	19.9 ± 13.1	16.6 ± 15.4	16.7 ± 12.2
**4 km·hr** ^**-1**^ **(3% gradient)**	0.95 ± 0.96	1.19 ± 0.95	1.01 ± 0.80	1.16 ± 0.81	21.4 ± 16.5	28.0 ± 18.0	24.3 ± 14.8	27.6 ± 14.5
**All activities**	**0.86 ± 0.82**	**0.69 ± 0.63**	**0.58 ± 0.56**	**0.59 ± 0.51**	**35.3 ± 30.8**	**33.0 ± 39.5**	**20.4 ± 14.3**	**21.0 ± 15.1**

## Discussion

Of the two accelerometers considered in this study, these data indicate that the GENEActiv device worn on either the upper arm or wrist provided the most valid (mean percentage error 3 & 4% for the upper arm and wrist, respectively) prediction of PAEE. For the GT3X+ the most appropriate anatomical location was on the wrist. There was a negligible difference in the strength of associations and error statistics for the GENEActiv device worn on the upper arm or wrist. Seemingly, incorporating raw acceleration signals as opposed to ‘arbitrary’ physical activity counts into linear regression models for the prediction of PAEE offered an improvement in the error of estimation for PAEE in MWUs (Fig 2; Table 3).

Considering physical inactivity has been associated with a cluster of metabolic abnormalities [25], it is surprising that relatively few studies have attempted to investigate the use of movement sensors in a population where self-report measures suggest that PA is substantially reduced. Despite employing some complex statistical modelling methods, previous studies have tended to use arbitrary ‘count’ values in the prediction of EE [21; 10] and also adopted a small selection of activities in their validation protocol [8; 26; 27]. The current study aimed to improve our understanding of accelerometer outputs and the prediction of PAEE in MWUs by incorporating raw acceleration values into linear regression models. Furthermore, participants performed a comprehensive wheelchair propulsion protocol which consisted of various velocities and gradients whereby the validity of these devices were assessed.

Previous research has provided encouragement for the wrist as the preferred anatomical location for previous generations of Actigraph accelerometers to predict V̇O_2_, explaining 44% [21] and 74% [10] of the variability in V̇O_2_, respectively. Off-the-shelf activity monitors incorporating manufacturer’s proprietary equations are unable to accurately predict EE in MWUs [27; 26]. As such, validation studies in this area have attempted to develop new predictive models. Washburn and Copay [21] generated a simple linear equation using counts·min^-1^ from the uniaxial CSA accelerometer over three propulsion velocities. Improvements in this prediction can be seen in the Garcia-Masso *et al*, [10] study, which used the GT3X tri-axial device and the acquisition of 1-s epochs to permit a feature extraction process which was incorporated into more complex multiple linear modelling (MLM). Previous work by our research group has demonstrated associations of *r* = 0.93 and *r* = 0.87 between counts·min^-1^ from the newest generation GT3X+ worn at the wrist and upper arm and PAEE during outdoor wheelchair propulsion [9]. One of the strengths of the present study was the controlled laboratory protocol adopted, being more comprehensive, including five extra activities, smaller increments in velocity (1km·hr^-1^ compared to 2km·hr^-1^) and various treadmill gradients. Consequently, weaker associations were observed between physical activity counts with criterion PAEE at the wrist and upper arm of *r* = 0.82 vs. *r* = 0.68. However, correlations between raw acceleration values expressed as SVM in *g*·minute^-1^ from the GENEActiv and criterion PAEE were similar to our previous field-based observations, at *r* = 0.88 and *r* = 0.87 for the wrist and upper arm, respectively. The GENEActiv-W has previously demonstrated excellent validity in able-bodied populations, displaying similar correlations to ours during a series of semi-structured laboratory and free living activities (left wrist vs. V̇O_2_, *r* = 0.86) [20].

Another strength of this present study was the comparison of two widely used accelerometry-based technologies to quantify PAEE. Specifically, by holding the anatomical location constant, variations in the strength of the relationships and error of estimate are inherent to the differences in the internal components, on-board filtering processes and outputs of each device [28]. To discard noise or movement artefacts unlikely to be representative of ‘human movement’, the GT3X+ has upper and lower bandwidth filters of 0.25 and 2.5Hz. These filters were designed for ambulation, based on the premise that acceleration frequencies arising from most human activities at the hip usually fall between this range. Bailey et al, [29] demonstrated that processed activity counts from a GT3X+ worn on the wrist are capable of distinguishing between tasks where upper extremities were used more intensively (e.g folding towels) than less intensively (e.g. writing). Whilst this protocol consisted of a comprehensive selection of upper extremity ADLs, no comparison was made to a PAEE criterion measurement, preventing the assessment of PAEE error. It is possible that these aforementioned filters are not suitable to capture movements at the wrist of MWUs, particularly during low-frequency ADLs as indicated by the sizeable over-estimation of 122% during the folding clothes task. However, excluding the folding clothes task from the analysis reduced the mean percentage error of estimate for the GT3X+-W to 0.4% during wheelchair propulsion at various speeds and gradients. Outputs from the GENEActiv are raw acceleration signals per unit time or epoch and are not subject to a tight bandwidth filter which may influence the prediction of PAEE at the wrist during certain activities. Whilst the GT3X+, and other commercially available monitors have the capability to report raw acceleration, the most common and easily accessible outputs from these devices are counts, which are influenced by the amplitude and frequency of acceleration. Physical activity counts have been shown to vary across devices and even within generations of the same type of device [30]. It is possible that the band-pass filtering and reporting of accelerometer outputs using ‘arbitrary’ units, which lack physical meaning, may be responsible for the differences in the error of estimation between the two devices (Fig 2; Table 3). As processing of raw data from the GT3X+ and other devices becomes available as standard then researchers can start to adopt an end-user practitioner approach to assessing the application and efficacy of these devices in the future.

Another explanation for the differences in associations for the two devices at the upper arm could be due to slight variations in their anatomical positioning and method of affixation. The GT3X+-UA worked loose during two trials, although these data were removed from subsequent analyses. It is possible that the secure attachment of the GENEActiv-UA provided by the medical tape minimised any movement inherent with the elastic belt of the GT3X+-UA. Whilst predictive models for the GENEActiv at the upper arm and wrist offer negligible bias in error prediction statistics and both under/over-predicted PAEE during three activities, the feasibility of attaching the GENEActiv to the upper arm during free living might be limited. Whereas the device worn on the wrist has a constant position, securely attached over the Styloid processes of the radius and ulna (worn like a watch). From a practical perspective, the GENEActiv worn on the wrist would be the preferred device/location for the accurate prediction of PAEE in MWUs. Accelerometers worn on the wrist are well tolerated and unobtrusive in this population and intuitively should not interfere with regular PA levels during free-living monitoring.

Considering the validity of an accelerometer based solely on the strength of its association to a criterion measure should be avoided as it does not indicate the agreement between the two variables [31]. Correlations are also dependent on the range of true quantity in the sample, seeing as our protocol had a wide selection of wheelchair propulsion velocities and gradients it is perhaps not surprising that this current study reported strong associations. A recent review on statistical considerations in the analysis of accelerometer data [22] advocated that it is useful for researchers to report measurement error. Mean percentage error of estimate (±SD) for the GT3X+-UA and GT3X+-W was 15 ± 45% and 14 ± 50%, compared to 3 ± 25% and 4 ± 26% for GENEActiv-UA and GENEActiv-W, respectively. Whilst our generated linear regression models for the GENEActiv demonstrated a relatively small bias, looking at MSE can be misleading as it is likely that under and over-estimations from different tasks cancel each other out.

An alternative is to look at mean absolute percentage error. Mean absolute percentage error ranged from 19–66%, 17–122%, 15–26% and 17–32% for the GT3X+-UA, GT3X+-W, GENEActiv-UA and GENEActiv-W, respectively. Previous research [27] attempted to develop new prediction models, using general and activity specific equations for an RT3 tri-axial accelerometer worn on the arm. The authors generated MLM’s using a training group of 19 participants and evaluated their performance on a smaller validation group (n = 4). The range of mean absolute percentage error using the general equation was 14–114% during an activity protocol that involved propulsion on a dynamometer, tiled floor and arm-ergometer exercise. This was similar to that of the GT3X+-W (17–122%). When looking solely at wheelchair propulsion, resting and deskwork using the activity specific equations, the mean absolute percentage error was reduced to 26% [27], which is slightly larger than the 20% and 21% for the GENEActiv upper arm and wrist for all activities included in our protocol.

The GT3X+-UA, GT3X+-W, GENEActiv-UA and GENEA-W monitors significantly over-predicted PAEE during rest by 0.31, 0.16, 0.32 and 0.54 kcal·min^-1^, respectively. This might have implications with the accurate prediction of PAEE during free-living. Garcia-Masso *et al*, [10] observed a significant over-prediction of estimated V̇O_2_ using a MLM from a device worn at the wrist during a lying down activity. Both monitors at the wrist also significantly over-estimate PAEE for the folding clothes activity. Nevertheless over-estimation of PAEE for light-intensity activities [32] or the inability to accurately describe the association between activity counts and the metabolic cost of certain lifestyle related activities [33] is a common observation when using accelerometers in the able-bodied physical activity monitoring literature. Even considering these limitations accelerometers are still widely used during cross-sectional and epidemiological physical activity research in ambulatory populations, observing similar issues in predicting PAEE when worn at the wrist in MWUs should not discourage their use in this population. Especially if there accuracy is better than current methods used to quantify PA in these cohorts. The relationships between raw acceleration at the upper arm and wrist (*r* = 0.87, *r* = 0.88) and criterion PAEE is better than the correlation between PARA-SCI scores and indirect calorimetry (*r* = 0.79) [4]. The authors found that this relationship was reduced and non-significant for low intensity activities (*r* = 0.27) and consequently the PARA-SCI scores under-reported the amount of time spent doing activities of low intensity by 10%. This self-report measure was instrumental in informing the most recent physical activity guidelines for adults with a chronic SCI [34]. The conversion of these scores using METS to predict EE would lead to a slight under-estimation. This is in contrast to our results and others [10], that accelerometers over-estimate PAEE for light intensity activities. It is of concern that error with monitoring sedentary behaviours may be exacerbated in a population whereby sedentary time may be elevated. One limitation of this study is that only one ADL was incorporated into the protocol whereby PAEE could be misclassified by the devices. Considering that 6–8 hrs of the day is spent in occupational tasks future work should incorporate more of these work-day tasks into laboratory validation protocols.

Limitations of accelerometers in the able-bodied literature are that outputs do not always reflect PAEE during walking on a slope [35] or during load carriage [36]. To the best of our knowledge, there is only one previous study looking at the validity of an activity monitor (SWA) in quantifying EE during wheelchair propulsion over differing gradients [37]. It is clear that proprietary algorithms used by the SWA over-estimate metabolic rate (MAE range; 24–126%) [26], but this overestimation and variability increased more when gradient was elevated, than when speed was increased [37]. This present study is the first to assess whether similar acceleration profiles with differing energy costs, such as changing gradient or load carriage, can be captured by new prediction models for tri-axial accelerometers in MWUs. There is a trend for all monitors to under-predict PAEE during propulsion on increasing gradients, and the GT3X+-W and GENEA-W significantly underestimated (-23 and -20%) PAEE during propulsion at 4km·hr^-1^ on a 3% gradient. Devices worn on the upper arm did not underestimate by the same magnitude as devices worn on the wrist during propulsion on differing gradients. It is possible that propulsion technique was modified, perhaps via an increase in flexion and extension of the shoulder to cope with the demands of uphill propulsion. Changes in propulsion patterns between conditions could be monitored using expensive motion analysis systems in future research studies.

Physical activity energy expenditure was not significantly different when an additional 8% of participant body mass was added to the chair during wheelchair propulsion at 4km·hr^-1^ (2.5 ± 0.7 vs. 2.4 ± 0.6 kcal·min^-1^). Sagawa *et al*., [38] noticed no significant effect of a 5 kg additional mass on EE or HR. It is plausible that a load attached to the wheelchair has a minimal impact on EE during propulsion on the flat unlike load carriage during ambulation. This may be because an 8% load is spread evenly across the axial and weight is supported in the vertical axis unlike walking. Importantly the MAE (Table 3) is not significantly different between propulsion at 4km·hr^-1^ and when an additional 8% of body mass is added for all monitors. Furthermore, each device displays relatively negligible biases during propulsion with additional weight.

The strengths of the present study are that RMR was accounted for and a comprehensive evaluation of two commonly used accelerometers, using a robust treadmill protocol with a variety of velocities and gradients was conducted. Previous studies have not controlled for RMR, which varies substantially between individuals with SCI, depending on level and completeness of lesion [39]. Previous validation work has often randomised the task order. With limited recovery time in between tasks it is therefore not always possible to exclude a carryover effect as a confounding variable, particularly when lower intensity activities follow higher intensity tasks. To avoid this, tasks were assigned in order of intensity as a method to prevent a carryover effect between trials. Fatigue seemingly had a minimal impact on our findings as we observed a strong linear relationship in physiological variables and accelerometer outputs across all tasks. These data would also suggest that assessing wheelchair propulsion using a controlled treadmill method is reflective of ‘real world’ propulsion. Despite using an independent group of participants and a portable metabolic analyser, PAEE during propulsion on the treadmill at 4km·hr^-1^ is identical to PAEE recorded during previous work involving propulsion on an athletics track at 4km·hr^-1^ (2.4 ± 0.6 vs. 2.4 ± 0.9 kcal·min^-1^) [9]. This is encouraging considering it has previously been recognized that treadmill walking/ running can affect gait mechanics and therefore may not reflect true metabolic costs of ambulation at a given speed over the ground [40]. Another considerable strength is that a ‘leave-one-out’ cross validation analysis of our generated prediction equations was conducted, an approach strongly advocated for future research whereby recruitment of participants with specific injury characteristics might be problematic. Reporting raw acceleration data in SI units (*g*·min^-1^) is a significant advantage as it allows easier comparison between devices and subsequently future research studies [14]. The developed linear regression model for the GENEActiv device could be utilised by other activity monitors that have the function or are capable of reporting raw acceleration values. However, the equivalency of raw outputs between monitors needs to be assessed in the future with regards to differences in dynamic sensing capacities (i.e. GENEActiv ± 8 *g* compared to ± 6 *g* for the GT3X+) or type of microelectromechanical systems (MES) sensor used.

This study demonstrated, for the first time, that the measurement of raw acceleration signals using the GENEActiv offered an improvement in the prediction of PAEE in MWUs. Specific on-board by-pass filtering methods intrinsic to the GT3X+ when reporting accelerometer data as activity counts appear to impact on the devices measurement sensitivity, particularly during low frequency movements (e.g. folding clothes). In keeping with the rapid development of activity monitoring over the past six years in ambulatory populations we expect the acquisition of raw data to become more prevalent in the prediction of PAEE during free-living.

## Supporting Information

S1 Dataset Influence of Accelerometer Type and Placement on Physical Activity Energy Expenditure Prediction in Manual Wheelchair Users Trial Dataset. The first two tabs display participant characteristics at rest and during peak oxygen consumption testing. Subsequent tabs contain accelerometer outputs and physiological variables for each participant during each task.
(XLSX)
